# Pharmacist Preceptor Exposure, Comfort and Awareness of Resources to Address the Social Determinants of Health—A Pilot Study

**DOI:** 10.3390/pharmacy11030083

**Published:** 2023-05-09

**Authors:** Tyler Marie Kiles, Karl R. Kodweis, Christa George, Chelsea Danielle Watts, Adalis Lock, Catherine Crill

**Affiliations:** 1 Department of Clinical Pharmacy and Translational Science, College of Pharmacy, The University of Tennessee Health Science Center, Memphis, TN 38163, USA; tkiles@uthsc.edu (T.M.K.); cgeorge1@uthsc.edu (C.G.);; 2 College of Pharmacy, The University of Tennessee Health Science Center, Memphis, TN 38163, USA

**Keywords:** preceptor development, social determinants of health

## Abstract

As preceptors are responsible for the experiential education of future pharmacists, it is important to assess understanding and identify knowledge gaps for preceptor development. The purpose of this pilot study was to assess the exposure to social determinants of health (SDOH), comfort in addressing social needs, and awareness of social resources among the preceptors at one college of pharmacy. A brief online survey was sent to all affiliated pharmacist preceptors with screening criteria for pharmacists who had regular one-on-one patient interactions. Of 166 preceptor respondents (response rate = 30.5%), 72 eligible preceptors completed the survey. Self-reported SDOH exposure increased along the educational continuum (with increasingly more emphasis from the didactic to experiential to residency). Preceptors who graduated after 2016, practiced in either community or clinic settings and served >50% of underserved patients were the most comfortable addressing social needs and the most aware of social resources. Preceptor understanding of SDOH has implications for their ability to educate future pharmacists. Colleges of pharmacy should evaluate practice site placement as well as preceptor knowledge and comfort in addressing social needs in order to ensure that all students are exposed to the SDOH throughout the continuum of learning. Best practices for up-skilling preceptors in this area should also be explored.

## 1. Introduction

The social determinants of health (SDOH) are nonmedical factors that affect a wide range of daily living, health risks, quality of life, and health outcomes [1,2,3]. For example, the physical and social environment in which people live, their income, and access to transportation and health services have implications for the health of individuals and communities [4]. The Centers for Disease Control and Prevention (CDC) defines the social determinants of health in five domains (Neighborhood and Built Environment, Health and Healthcare, Social and Community Context, Education Access and Quality, and Economic Stability) [1] and pharmacists have unique roles and responsibilities in addressing the SDOH in each of these domains [5]. The Accreditation Council for Pharmacy Education (ACPE) and Center for the Advancement of Pharmacy Education (CAPE) educational outcomes include Standard 3.5 guidance for pharmacy schools to develop graduates who can recognize the social determinants of health to diminish disparities and inequities in access to quality care [6,7].

Considering that it is important for pharmacists to understand and address social risk factors in practice, student pharmacists must be exposed to the SDOH along the continuum of learning, and incorporating the SDOH into both the didactic and experiential curriculum is essential [8]. Through introductory and advanced pharmacy practice experiences (IPPEs and APPEs), students are able to put into practice what they learn in the classroom under the guidance and supervision of a pharmacist preceptor. Effective preceptors must have expertise in their teaching area and serve as a guide/mentor for students as they develop competencies for pharmacy practice [6,9]. Through observation, guidance, and mentorship, preceptors can truly have an impact on student learning and future practice.

However, the understanding of the SDOH and commitment to addressing health equity was only recently added to pharmacy accreditation standards in 2016; therefore, it is unknown if active preceptors who matriculated in pharmacy school before 2016 have adequate exposure or knowledge to effectively develop and mentor future pharmacists in this area. Previous curricular requirements related to patient care in this realm related to cultural competency—an appreciation for diverse beliefs, values and behaviors as a way to tailor patient care based on cultural, social and linguistic needs [10,11,12]—however, cultural competency training alone fails to address the structural and systemic inequities that patients and communities may face in interfacing with the healthcare system. Although having cultural intelligence and demonstrating cultural competency can be very valuable to establishing a patient-provider relationship, how preceptors and practicing pharmacists understand and intervene on the social determinants of health has not been thoroughly explored. 

Research has shown that among healthcare providers, comfort with regard to addressing the social determinants of health is low at baseline, and there is scarce literature about pharmacists specifically. One study by Schickedanz and colleagues found that among clinicians (including physicians, social workers, nurses and pharmacists) in a large integrated health system in California, only 41% felt confident in their ability to address social needs [13]. Another small study of pharmacy personnel in a retail chain pharmacy in the Mid-South region of the United States found that the pharmacists and technicians lacked confidence and comfort when screening for social needs [14]. However, in a study by Li et al., pharmacist preceptors expressed confidence in discussing factors underlying health disparities such as access, socioeconomic status, environment and racial/ethnic disparities with experiential learners [15].

There is increasing support and opportunity for pharmacists to intervene on the SDOH [16,17] and this requires an awareness of social resources. While there has been discussion about how pharmacy schools can incorporate SDOH in pharmacy curricula [8,18,19,20,21,22], there is little investigation of the previous exposure to and knowledge of SDOH among pharmacist preceptors who graduated before the new standards were introduced. It is unknown whether pharmacist preceptors are comfortable addressing social needs or are aware of social resources which has implications for how effectively they are able to educate our students. As preceptors are responsible for the experiential education of future pharmacists, it is important to assess understanding and address knowledge gaps related to the SDOH among this population. 

The purpose of this pilot study was to assess the exposure, comfort, and awareness of social resources among preceptors at the University of Tennessee Health Science Center (UTHSC) College of Pharmacy to guide the creation of targeted preceptor development. 

## 2. Materials and Methods

An electronic survey was sent to all pharmacist preceptors affiliated with the UTHSC College of Pharmacy (*n* = 545) across the state of Tennessee. The brief online survey collected demographic information and assessed preceptor exposure, comfort, and awareness of social resources using Likert-scale questions. As no standardized assessments are available for knowledge and application of the SDOH, the survey was developed using relevant literature [1,2] and validated by expert opinion. The survey was pretested with three pharmacist preceptors and based on their feedback, the phrasing of two questions was minimally revised to improve clarity. 

The investigators of this study aimed to develop future pharmacists capable of conducting in-depth social needs interactions while providing substantial patient care—therefore, in this exploratory analysis, initial screening criteria were imposed to specifically investigate preceptors with significant patient care responsibilities and opportunities for one-on-one patient interactions. The first few questions of the survey served to screen for preceptors who had regular opportunities to address the SDOH in one-on-one patient care interactions. Therefore, respondents were excluded from the analysis if their patient interaction was limited to less than 10 patients per day, if they did not spend time independently talking to patients outside of an interprofessional team setting, and if they could not spend at least 5 min talking to a patient. Participants meeting the aforementioned criteria were screened out using survey logic and, thus, could not complete the remainder of the survey. 

After pre-screening, eligible survey participants (*n* = 101) were presented with a brief description of the social determinants of health as depicted by the Kaiser Family Foundation to ensure an understanding of the terminology [23]. To capture exposure to SDOH, participants were asked to reflect and select the degree to which each domain was emphasized in their didactic, experiential, and post-graduate curricula. They were also asked to rank their current level of comfort addressing certain social risk factors (food insecurity, housing instability, lack of transportation, financial instability or unemployment, and interpersonal violence) and awareness of various social resources available in their communities. Demographic information collected included: degree of training, graduation year, practice setting, and patient demographics, as well as respondent age, gender, and race/ethnicity. Preceptor geographic location was not captured to protect anonymity. The UTHSC Institutional Review Board approved this study.

Investigators have reported descriptive statistics. Due to the small sample size of various demographics, extensive statistical sub-analyses were not conducted; however, the survey respondent characteristics were explored. For analysis of comfort, a composite score was calculated for several respondent characteristics. The comfort composite score was calculated as such: the median comfort level on the 5-point Likert scale (for five social risk factors, one from each SDOH domain) was summed and averaged (out of 25 possible total) for analysis and comparison. On a scale of 0 to 1, a composite score of 1 would indicate extreme comfort in addressing social risk factors. Exposure and awareness results are presented in the aggregate to describe general trends and observations.

## 3. Results

Out of 545 active preceptors, a total of 166 preceptors responded to the survey (response rate = 30.5%) and 65 responses were excluded from the analysis based on screening questions. Of 101 eligible participants, 72 preceptors completed the survey (71.3% completion rate). The full demographic breakdown for the eligible participants can be found in Table 1.

### 3.1. Exposure to Social Determinants of Health in Pharmacy Education

Preceptors were asked to recall whether the SDOH were taught and the degree to which each domain was emphasized in their didactic, experiential, and post-graduating education and training. Sixty-five percent (*n* = 47), 68% (*n* = 49), and 45% (*n* = 22) of participants reported that the SDOH were not taught at all in their didactic, experiential, and residency training, respectively. Of those responding ‘no’ to recalling if SDOH were taught in their pharmacy training, 79% were preceptors who graduated before 2016.

**Figure 1:**
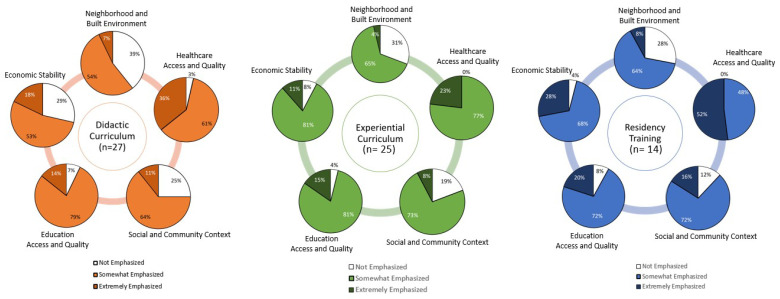
Pharmacist Preceptors’ Perceptions of the Degree of Emphasis of Social Determinants of Health in Pharmacy Curriculum.

Along the pharmacy training continuum, emphasis on the SDOH increased from didactic to experiential to residency. Across all five SDOH domains, respondents indicated a greater emphasis on SDOH in experiential training vs. didactic education. For example, reported emphasis on “Economic Stability” was more emphasized in the experiential setting (92%) than in didactic education (71%). The “Neighborhood and Built Environment” domain was the least emphasized of the five domains across all training levels. A full breakdown of the reported curricular emphasis of all five SDOH domains across the different levels of training can be seen in Figure 1.

### 3.2. Comfort Addressing Social Needs

**Figure 2:**
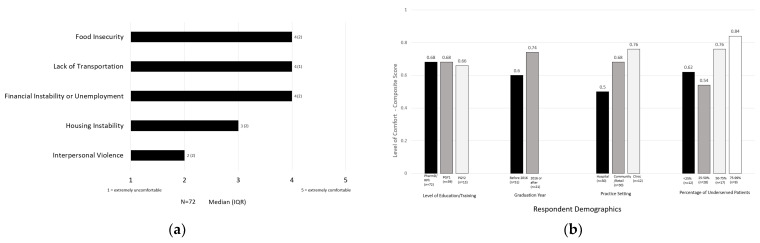
(**a**) Aggregate Pharmacist Preceptor Comfort Addressing Social Needs; (**b**) Pharmacist Preceptor Comfort Composite Scores by Respondent Demographics.

Preceptors’ responses regarding their comfort with addressing social needs can be found in Figure 2. The median responses are summarized in the aggregate (Figure 2a) and subgroup analyses for graduation year, practice setting, and percentage of the patient population described as underserved are depicted in Figure 2b.

The preceptors in this sample reported the highest level of comfort in addressing food insecurity, financial instability, and transportation, and were less comfortable addressing housing instability and interpersonal violence. Preceptors who graduated after 2016 (when ACPE Standards were updated) were more comfortable addressing social needs than pharmacists who graduated before 2016 (composite scores: 0.74 vs. 0.60, respectively). Preceptors who practiced in the community (composite score: 0.76) and clinic settings (composite score: 0.68) were more comfortable addressing social needs than respondents precepting in hospital settings (composite score: 0.5). Pharmacists who served a larger proportion of underserved patients (>75%) had the highest comfort with addressing social needs.

### 3.3. Awareness of Social Resources

**Figure 3:**
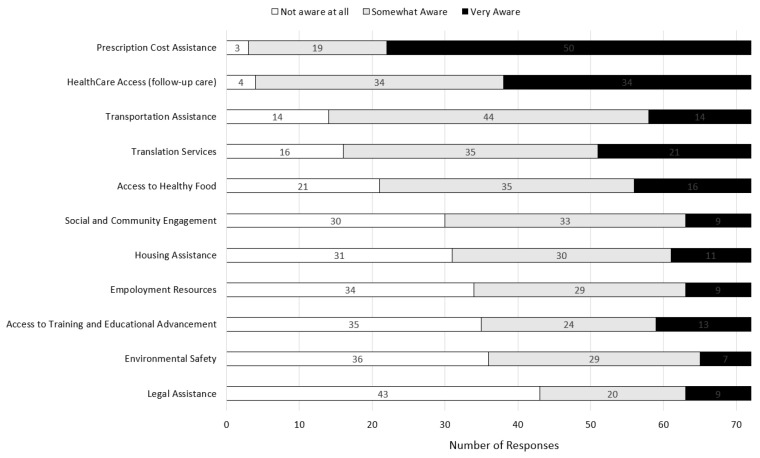
Pharmacist Preceptor Awareness of Social Resources.

Preceptors’ responses regarding their awareness of resources to address the social determinants of health are displayed in Figure 3.

Preceptors reported the highest level of awareness of prescription cost assistance (*n* = 50, 69.4% “very aware”) and healthcare access. Conversely, preceptors reported the lowest awareness of resources focusing on legal assistance (*n* = 43, 59.7% “not aware at all”), environmental safety, training and educational advancement, and employment resources. Preceptors reported a moderate level of awareness of the other resources assessed (see Figure 3).

The breakdown of respondent characteristics (by year graduated, practice setting, and percentage of the patient population described as underserved) is summarized in Appendix A Table A1. In general, preceptors in the hospital setting were less aware of social resources compared to the others, and preceptors in the clinic setting were the most aware of social resources overall. Those who served higher percentages of underserved patients also reported a greater awareness of nearly all resources. Across all resources, those who graduated before 2016 were more likely to select “not aware at all” than those who graduated after 2016. 

## 4. Discussion

In this exploratory study of pharmacist preceptors who regularly engage with patients in practice, there is limited exposure, comfort, and awareness of resources to address the SDOH. The results of this pilot study suggest that there is an opportunity for preceptor development in this area to ensure our pharmacy graduates are being prepared for practice readiness upon graduation.

Most preceptors in this study responded ‘no’ when asked if the SDOH was taught in pharmacy training, and this was heavily influenced by the high number of preceptors who were pre-2016 graduates in our sample. In this study, the preceptors in this sample who graduated after 2016 reported higher levels of comfort with addressing social needs and awareness of social resources. While there is the chance that this observation may be affected by the limited sample size or recall bias, it is also plausible that because the SDOH was not previously an accreditation requirement, more experienced preceptors may have had limited formal didactic exposure. This deserves further study, for in a previous investigation within a community pharmacy chain, newer practitioners reported receiving more training, while preceptors who had been practicing for >5 years reported more comfort in conducting SDOH screenings [9]. Further investigation is necessary to determine whether reported comfort in this area translates to the effective education of future pharmacists, or corresponds to increased self-efficacy [19,24] or consistent action or appropriate intervention on social needs for patients. Of note, in those who did recall learning about the SDOH, Neighborhood/Built Environment was the least emphasized. This is consistent with a review of the literature on SDOH in pharmacy education which found that this SDOH domain was least emphasized through published active learning techniques in pharmacy education [18].

This pilot study showed that the pharmacists in our sample had a higher level of comfort and awareness of resources to address social risk needs most directly related to pharmacy practice (such as transportation and prescription cost assistance) and were less comfortable or aware of resources not directly applicable to pharmacy practice (for example, legal assistance, interpersonal violence, and educational training and advancement). Awareness of a myriad of social resources is important for healthcare providers to provide holistic patient care; however, it is also important for the profession to recognize the ethical dilemma of asking patients about social risk factors without the ability to address them [25]. While pharmacists may not have the capacity or training to directly address all social needs in practice, pharmacists may serve as a referral source to the community or social connections [5]. Appropriate training may educate preceptors to create lists of community-specific resources or to effectively utilize other networks, such as FindHelp.org, a comprehensive database of social assistance programs based on zip code for various social needs, such as food, housing, education, employment, or legal aid. Pharmacists may also leverage interprofessional collaborations with social work, care navigators, or community health workers to assist patients with social services [5]. More studies must be conducted in this area to determine the most appropriate interventions for pharmacists to make related to the SDOH and to design and develop preceptor education accordingly. 

This study adds to the literature around the SDOH in pharmacy education, in that these participants described an increase in SDOH exposure along the continuum of pharmacy education (from didactic to experiential to post-graduate training). Although exposure to SDOH in the didactic curriculum is still ongoing [21], as colleges of pharmacy endeavor to further integrate the SDOH as an aspect of pharmacy practice, emphasis in the experiential setting may be a natural fit. However, experiential learning about the SDOH may be influenced by the types of practice sites available and the knowledge and experience of the preceptors that students are assigned. This study revealed that there is variation in preceptor comfort addressing the SDOH by years of experience, practice site, and patient demographics.

To ensure that all students are exposed to the SDOH in experiential learning, colleges and schools of pharmacy experiential offices should continue to work to place students at practice sites that involve people from a range of different social backgrounds. To evaluate whether students are experiencing significant exposure to the SDOH in experiential settings, preceptor knowledge of the SDOH may be collected and evaluated for each site, through survey assessments such as this or during routine site visits. Alternatively, colleges may consider adding questions to formal student evaluations of preceptors and sites to determine the rotations/preceptors that provided students with significant SDOH exposure/interventions. If feasible from a scheduling standpoint, colleges could elect to assign students to at least a minimum number of these rotations and incorporate student reflection on the SDOH as part of that experience.

To ensure students are adequately prepared by preceptors to address SDOH in practice, experiential offices should also endeavor to profile their preceptors’ knowledge about SDOH and educate them as necessary through preceptor development. Currently, to serve as a preceptor with UTHSC, pharmacists must have credentials (residency training) and/or sufficient experience in the care and management of patients in the patient care setting of their rotation category (i.e., community pharmacy practice, ambulatory care, general medicine, specialty pharmacy practice area, etc.), must maintain licensure in the state of in which they practice, complete a college preceptor orientation, and complete any mandatory preceptor development when assigned. Targeted preceptor development may be considered. For example, with the information from this study, UTHSC College of Pharmacy preceptor development may target pharmacists practicing for >10 years, hospital pharmacists, or those who serve <50% underserved patients to increase their knowledge around the SDOH. The preceptors in this sample did not express comfort in addressing interpersonal violence or housing instability. These social needs are potential targets for preceptor development programming as well. 

A previous study suggested that a 1 h continuing education training may improve community pharmacy personnel knowledge and comfort in engaging patients about social needs [9]. In this study; however, the authors also suggested that active learning techniques, simulation-based learning, and practice scenarios would be most effective for training practicing pharmacists to engage with patients about social risk factors [14]. Alternatively, pharmacy students may also be leveraged to educate preceptors on SDOH through innovative educational models. For example, one study incorporated student-delivered preceptor development during APPEs [26]. Experiential learning faculty trained students to educate their preceptors from a menu of topics through one-to-one discussion during the students’ rotation. Students used one-page teaching sheets to facilitate brief (<10 min) discussions with their preceptor. At the end of the study, preceptors preferred the student-delivered preceptor development over other delivery methods and documentation of preceptor development improved. On the other hand, students themselves could serve in precepting roles during APPEs. For example, one study evaluated student perceptions of their experiences of non-traditional student-preceptor models: peer-assisted learning (PAL), near-peer teaching (NPT), and co-precepting (CoP) [27]. During APPEs where PAL and/or NPT were used, students reported feeling supported through the fostering of collaborative learning and overall, students reported this way of learning was an enhanced approach to patient care and professional practice.

There are several limitations to this research. For one, responses may be subject to recall bias and other limitations related to self-report. Additionally, this pilot study of active preceptors affiliated with one university screened out pharmacists who did not see >10 patients per day. This was our population of interest for targeted development; however, these criteria may have missed preceptors who are regularly involved in patient care, but see, for example, 6–8 patients per day. Future studies should investigate the broader landscape of pharmacist preceptor knowledge in this area to determine overall areas for improvement for the academy. This sample was also predominately white and female. While this is consistent with the demographics of our college’s preceptor pool, it is not consistent with the demographics of the patient population within the state of TN which is 16.6% Black and 4.6% Hispanic/Latino [28]. In Tennessee, there are also significant patient demographic differences (race/ethnicity, rural/urban) depending on location within the state. Further investigation should explore if there are racial/ethnic, gender, geographic, training or site-specific, or other demographic differences in preceptor exposure, comfort, or awareness of the SDOH. For example, training provided outside of pharmacy education, (such as through on-the-job training, employer SDOH training modules or certificate programs) was not investigated in this study and warrants exploration. Due to differences in sample size, we were unable to conduct extensive statistical analysis. Future studies with larger populations and validated survey instruments, as well as qualitative inquiry, will shed more light on this area. 

For the academy, it is important to note that exposure, awareness, or self-reported comfort does not necessarily translate to ability and action on the SDOH. Future research should investigate facilitators that empower pharmacists to make a meaningful impact in this area. In addition to the management of downstream individual patient social needs, pharmacists must also be trained to address upstream factors related to the SDOH. The forces and systems that shape the conditions of daily life include economic policies, social norms, and systems. These structural factors cannot be overlooked when designing and implementing educational strategies pertaining to the SDOH in pharmacy [5,29].

## 5. Conclusions

Preceptor understanding of SDOH has implications for their ability to educate future pharmacists. Colleges of pharmacy should evaluate practice site placement as well as preceptor knowledge and comfort in addressing social needs in order to ensure that all students are exposed to the SDOH throughout the continuum of learning. Best practices for up-skilling preceptors in this area should also be explored.

## Figures and Tables

**Table 1:** Pharmacist Preceptor SDOH Survey Respondent Demographics.

Age, *n* (%)	All (*n* = 72)
25–39 years old	50 (69.4)
40–59 years old	21 (29.2)
>60 years old	1 (1.4)
Gender, *n* (%)	
Female	52 (72.2)
Male	19 (26.4)
Prefer not to disclose	1 (1.4)
Race/Ethnicity, *n* (%)	
White/Caucasian	62 (86.1)
Black/African American	4 (5.6)
Asian/Pacific Islander	2 (2.8)
Multiracial	2 (2.8)
Prefer not to disclose	2 (2.8)
Graduation Year, *n* (%)	
Before 2016	51 (70.8)
After 2016	21 (29.2)
Level of Education/Training, *n* (%)	
PharmD, RPh	72 (100.0)
PGY1 Residency *	39 (50.0)
PGY2 Residency *	14 (19.4)
Practice Area, *n* (%)	
Community or Retail	30 (41.7)
Hospital	30 (41.7)
Clinic	12 (16.7)
Percent of Underserved Patients, *n* (%)	
<25% Underserved	12 (16.7)
25–50% Underserved	27 (37.5)
50–75% Underserved	17 (23.6)
75–100% Underserved	9 (12.5)
Unspecified	7 (9.7)

* PGY1—first postgraduate year; PGY2-s postgraduate year.

## Data Availability

The data presented in this study are available on request from the corresponding author. The data are not publicly available due to IRB limitations.

## Appendix A

**Table A1:** Awareness of community resources to address the social determinants of health, stratified by graduation year, practice setting, and the percentage of the underserved patient population.

Item	Awareness Level	Aggregate *n* (%)	Graduation Year		Practice Setting			% Underserved			
			Before 2016 *n* (%)	After 2016 *n* (%)	Hospital *n* (%)	Community/Retail *n* (%)	Clinic *n* (%)	<25% *n* (%)	25–50% *n* (%)	50–75% *n* (%)	75–100% *n* (%)
Prescription cost assistance	Not at all aware	3 (4.2)	3 (5.9)	0 0.0)	1 (3.3)	2 (6.7)	0 (0.0)	0 (0.0)	1 (3.7)	0 (0.0)	0 (0.0)
	Somewhat aware	19 (26.4)	15 (29.4)	4 (19.0)	9 (30.0)	8 (26.7)	2 (16.7)	5 (41.7)	9 (33.3)	3 (17.6)	0 (0.0)
	Very aware	50 (69.4)	33 (64.7)	17 (81.0)	20 (66.7)	20 (66.7)	10 (83.3)	7 (58.3)	17 (63.0)	14 (82.4)	9 (100.0)
Healthcare Access	Not at all aware	4 (5.6)	4 (7.8)	0 (0.0)	1 (3.3)	3 (10.0)	0 (0.0)	1 (8.3)	1 (3.7)	0 (0.0)	0 (0.0)
	Somewhat aware	34 (47.2)	26 (51.0)	8 (38.1)	17 (56.7)	14 (46.7)	3 (25.0)	6 (50.0)	15 (55.6)	8 (47.1)	2 (22.2)
	Very aware	34 (47.2)	21 (41.2)	13 (61.9)	12 (40.0)	13 (43.3)	9 (75.0)	5 (41.7)	11 (40.7)	9 (52.9)	7 (77.7)
Access to healthy food	Not at all aware	21 (29.20	15 (29.4)	6 (28.6)	14 (46.7)	7 (23.3)	0 (0.0)	2 (16.7)	12 (44.4)	4 (23.5)	2 (22.2)
	Somewhat aware	35 (48.6)	28 (54.9)	7 (33.3)	14 (46.7)	11 (36.7)	10 (83.3)	8 (66.7)	10 (37.0)	7 (41.2)	4 (44.4)
	Very aware	16 (22.2)	8 (15.7)	8 (38.1)	2 (6.7)	12 (0.0)	2 (16.7)	2 (16.7)	5 (18.5)	6 (35.3)	3 (33.3)
Housing assistance	Not at all aware	31 (43.1)	24 (47.1)	7 (33.3)	17 (56.7)	11 (36.7)	3 (25.0)	5 (41.7)	14 (51.9)	6 (35.3)	2 (22.2)
	Somewhat aware	30 (41.7)	22 (43.1)	8 (38.1)	11 (36.7)	12 (40.0)	7 (58.3)	6 (50.0)	9 (33.3)	7 (41.2)	5 (55.5)
	Very aware	11 (15.3)	5 (9.8)	6 (28.6)	2 (6.7)	7 (23.3)	2 (16.7)	1 (8.3)	4 (14.8)	4 (23.5)	2 (22.2)
Transportation assistance	Not at all aware	14 (19.4)	11 (21.6)	3 (14.3)	9 30.0)	5 (16.7)	0 (0.0)	3 (25.0)	7 (25.9)	0 (0.0)	2 (22.2)
	Somewhat aware	44 (61.1)	32 (62.7)	12 (57.1)	19 (63.3)	16 (53.3)	9 (75.0)	8 (66.7)	17 (63.0)	12 (70.6)	3 (33.3)
	Very aware	14 (19.4)	8 (15.7)	6 (28.6)	2 (6.7)	9 (30.0)	3 (25.0)	1 (8.3)	3 (11.1)	5 (29.4)	4 (44.4)
Access to training and educational advancement	Not at all aware	35 (48.6)	27 (52.9)	8 (38.1)	19 (63.3)	9 (30.0)	7 (58.3)	5 (41.7)	17 (63.0)	6 (35.3)	2 (22.2)
	Somewhat aware	24 (33.3)	16 (31.4)	8 (38.1)	8 (26.7)	14 (46.7)	2 (16.7)	6 (50.0)	8 (29.6)	6 (35.3)	2 (22.2)
	Very aware	13 (18.1)	8 (15.7)	5 (23.8)	3 (10.0)	7 (23.3)	3 (25.0)	1 (8.3)	2 (7.4)	5 (29.4)	5 (55.5)
Employment resources	Not at all aware	34 (47.2)	26 (51.0)	8 (38.1)	18 (60.0)	11 (36.7)	5 (41.7)	7 (58.3)	13 (48.1)	8 (47.1)	2 (22.2)
	Somewhat aware	29 (40.3)	20 (39.2)	9 (42.9)	11 (36.7)	13 (43.3)	5 (41.7)	5 (41.7)	11 (40.7)	5 (29.4)	5 (55.5)
	Very aware	9 (12.5)	5 (9.8)	4 (19.0)	1 (3.3)	6 (20.0)	2 (16.7)	0 (0.0)	3 (11.1)	4 (23.5)	2 (22.2)
Legal assistance	Not at all aware	43 (59.7)	31 (60.8)	12 (57.1)	23 (76.7)	12 (40.0)	8 (66.7)	7 (58.3)	19 (70.4)	9 (52.9)	5 (55.5)
	Somewhat aware	20 (27.8)	15 (29.4)	5 (23.8)	6 (20.0)	11 (36.7)	3 (25.0)	4 (33.3)	5 (18.5)	4 (23.5)	3 (33.3)
	Very aware	9 (12.5)	5 (9.8)	4 (19.0)	1 (3.3)	7 (23.3)	1 (8.3)	1 (8.3)	3 (11.1)	4 (23.5)	1 (11.1)
Translation services/ Language assistance	Not at all aware	16 (22.2)	12 (23.5)	4 (19.0)	7 (23.3)	8 (26.7)	1 (8.3)	1 (8.3)	9 (33.3)	2 (11.8)	3 (33.3)
	Somewhat aware	35 (48.6)	24 (47.1)	11 (52.4)	16 (53.3)	14 (46.7)	5 (41.7)	9 (75.0)	13 (48.1)	6 (35.3)	2 (22.2)
	Very aware	21 (29.2)	15 (29.4)	6 (28.6)	7 (23.3)	8 (26.7)	6 (50.0)	2 (16.7)	5 (18.5)	9 (52.9)	4 (44.4)
Environmental safety	Not at all aware	36 (50.0)	28 (54.9)	8 (38.1)	19 (63.3)	12 (40.0)	5 (41.7)	4 (33.3)	18 (66.7)	8 (47.1)	4 (44.4)
	Somewhat aware	29 (40.3)	19 (37.3)	10 (47.6)	10 (33.3)	13 (43.3)	6 (50.0)	8 (66.7)	7 (25.9)	5 (29.4)	4 (44.4)
	Very aware	7 (9.7)	4 (7.8)	3 (14.3)	1 (3.3)	5 (16.7)	1 (8.3)	0 (0.0)	2 (7.4)	4 (23.5)	1 (11.1)
Social/community engagement	Not at all aware	30 (41.7)	22 (43.1)	8 (38.1)	18 (60.0)	7 (23.3)	5 (41.7)	4 (33.3)	13 (48.1)	8 (47.1)	4 (44.4)
	Somewhat aware	33 (45.8)	24 (47.1)	9 (42.9)	11 (36.7)	17 (56.7)	5 (41.7)	7 (58.3)	12 (44.4)	5 (29.4)	3 (33.3)
	Very aware	9 (12.5)	5 (9.8)	4 (19.0)	1 (3.3)	6 (20.0)	2 (16.7)	1 (8.3)	2 (7.4)	4 (23.5)	2 (22.2)
